# 

**Published:** 1860-12

**Authors:** 


### CONTENTS.

### ORIGINAL COMMUNICATIONS.

PAGE.

ARTICLE I.—Cancer of the Uterus; Compression of the Uterus; Consecutive Hydronephrosis; Suppression of the Urinary Secretion; Nervous Complications. By M. Aaran, at the Hotel-Dien. (Translated from the Gazette des Hospitaux.) By ROBERT BATTEY, M. D., Rome, Ga., ........ 191

ARTICLE II.—Children—Their requirements, Moral and Physical, Essential to a Healthful Development—Influences which have a Salutary or Baneful Effect, etc., ........ 202

ARTICLE III.—Hints to Consumptives. By F. W. HEMMING, M.D. Savannah, Ga., ........ 208

ARTICLE IV.—Review by V. H. TALLIAFERRO, M. D. of "The Anatomy and Physiology of the Placenta; The Connection of the Nervous Centres of Animal and Organic Life." By JOHN O'REILLY, M. D., ........ 217

### SELECTIONS.

Selected cases, with Medical and Therapeutical Observations. By F. PEYRE PORCHER, M. D., Physician to the Marine Hospital, Charleston, S. C., ........ 225

### EDITORIAL AND MISCELLANEOUS.

Jaundice, ........ 246

A Stir about Female Physicians, ........ 250

Year Book of American Contributions to Medical Science and Literature, ........ 250

Meteorological Table, ........ 252

